# The Cost-Effectiveness of and Adherence to Disease-Modifying Antirheumatic Drug (DMARD) Therapy in Rheumatoid Arthritis Patients in a Tertiary Care Teaching Hospital in Uttarakhand, India

**DOI:** 10.7759/cureus.34664

**Published:** 2023-02-06

**Authors:** Gauri Mittal, Manisha Bisht, Venkatesh S Pai, Shailendra Handu

**Affiliations:** 1 Pharmacology, All India Institute of Medical Sciences, Rishikesh, Rishikesh, IND; 2 Internal Medicine and Clinical Immunology/Rheumatology, All India Institute of Medical Sciences, Rishikesh, Rishikesh, IND

**Keywords:** disease activity score (das28), methotrexate, quality of life (whoqol-bref questionnaire), dmard therapy, rheumatoid arthritis

## Abstract

Objective

This study was conducted with the aim of evaluating the cost-effectiveness of and adherence to treatment in patients on disease-modifying antirheumatic drug (DMARD) therapy for rheumatoid arthritis (RA) in a tertiary care teaching hospital in Uttarakhand, India.

Methodology

This prospective observational study was conducted on 150 rheumatoid arthritis patients presenting to the Rheumatology Outpatient Department (OPD) receiving DMARD therapy (approval number AIIMS/IEC/18/160). The patients were followed up for an average of 10.7 weeks and received drugs in four regimens with methotrexate (MTX) (Regimen 1) having the least contribution with a mean of 46.05 Rs, methotrexate + hydroxychloroquine (MTX + HCQ) (Regimen 2) with 174.15 Rs, methotrexate + hydroxychloroquine + leflunomide (MTX + HCQ + Lef) (Regimen 3) with Rs 371.70, and methotrexate + hydroxychloroquine + leflunomide + biological DMARD adalimumab (MTX + HCQ + Lef + bDMARD adalimumab) (Regimen 4) with 17,349.4 Rs. The cost of drug therapy was assessed by calculating the cost of therapy per month for each patient, and adherence was assessed using the Morisky-Green-Levine Scale (MGLS) at the follow-up visit.

Results

The overall mean cost of DMARD treatment was 205.81 Rs. The overall DMARD therapy cost-effectiveness was Rs 878.14 for a unit change of Disease Activity Score (DAS28). The most cost-effective treatment came out to be Regimen 1 with the least cost of 290.9 Rs for a unit change of DAS28, and the least cost-effective was Regimen 4 with 65,661.8 Rs for a unit change of DAS28. At follow-up, among all subjects of the study, 49 (32.7%) subjects showed high adherence, 71 (47.3%) subjects showed medium adherence, and 30 (20%) subjects showed low adherence. Accordingly, the maximum number of participants fell in the category of medium adherence, i.e., 71 (47.4%).

Conclusion

Our study concluded that the cost burden varied according to the number of DMARDs being given to the patient. The double-drug therapy of methotrexate + hydroxychloroquine had a maximum “high adherence.” On a whole, the majority of patients had “medium adherence” to therapy.

## Introduction

Rheumatoid arthritis (RA) is a systemic autoimmune disease that attacks the joints of the skeletal system. The most commonly affected joints are the small joints of the hands, wrists, and knees, causing stiffness and painful swelling in the affected parts of the body. It usually follows a symmetric, multiple-joint pattern. The lining of the affected joint becomes inflamed, damaging it over a period of time, causing chronic pain and deformity [1]. RA patients are recommended to be treated with disease-modifying antirheumatic drugs (DMARDs) within three months of diagnosis, as per guidelines [2]. The standardized mortality ratio in patients with RA has been found to be 2.26, meaning a person having RA has two times more probability of dying at the same age compared with an average person not having RA in the population [3].

The current treatment of RA relies on the augmentation and blockade of different aspects of the immune response through drug administration. Curing RA is not possible with the remedies at hand today, but it is possible to achieve remission in some patients. Drug treatment ranges from nonsteroidal anti-inflammatories (NSAIDs) to DMARDs, to the specific blockade of certain immune cells or pro-inflammatory mediators using biologics [4]. The ratio of the annual direct costs of treatment when compared between those having RA and those having osteoarthritis (OA) is nearly twice, while indirect costs for RA are also significantly different, being five times more for RA compared with OA, the reason being decreased productivity [5]. RA, like chronic illnesses, needs long-term use of drugs. However, because many patients do not adhere to the medication plan as prescribed, the full benefits of the drug treatment often remain unrealized [6]. Poor adherence leads to poor clinical outcomes and increased healthcare utilization and costs [7]. Advanced age has been observed to be associated with better drug adherence [8]. Some more issues affecting medication adherence include efficacy, doctor-patient relationship, and social support in patients with RA [9]. Hence, this study was planned to assess adherence to treatment and the pharmacoeconomic aspect in patients with rheumatoid arthritis.

## Materials and methods

This prospective observational study was carried out in the Department of Pharmacology in a tertiary care institute over a period of one year after approval from the Institutional Ethics Committee (All India Institute of Medical Sciences (AIIMS), Rishikesh) (approval number AIIMS/IEC/18/160). Our study followed the principles of the Declaration of Helsinki. Subjects were recruited from patients presenting to the Rheumatology Outpatient Department (OPD) with a primary diagnosis of RA after obtaining written informed consent. Inclusion criteria were all new and previously diagnosed patients with rheumatoid arthritis based on the American College of Rheumatology (ACR) 2010 diagnostic criteria of either sex. Patients excluded from this study included those affected with arthritis due to reasons other than RA, such as vasculitis, polymyalgia rheumatica, spondyloarthropathies (reactive arthritis, ankylosing spondylitis, and psoriatic arthritis), bacterial arthritis, and fibromyalgia.

On the basis of DMARD therapy being received by the patients, they were divided into different groups: Regimen 1, monotherapy with one DMARD (methotrexate (MTX)); Regimen 2, double DMARD therapy or two DMARD therapy (methotrexate + hydroxychloroquine (MTX + HCQ)); Regimen 3, triple DMARD therapy or three DMARD therapy (methotrexate + hydroxychloroquine + leflunomide (MTX + HCQ + Lef)); and Regimen 4, >3 DMARD therapy (MTX + HCQ + Lef + bDMARD adalimumab). Patients were assessed at baseline and after the follow-up visit as per the clinician’s discretion (varying from 7 to 12 weeks). Treatment response was recorded at the baseline and follow-up visit based on the Disease Activity Score (DAS28) criteria, which comprises a number of tender joints, swollen joints, erythrocyte sedimentation rate (ESR), and “patient global health” score [10].

The medication cost of DMARD therapy was analyzed by calculating the cost of therapy per month for each patient by taking the prices from the Bureau of Pharma Public Sector Undertakings of India (BPPI), Department of Pharmaceuticals, Government of India, for all the DMARDs, except the biological drug for which the price from Cadila Healthcare Ltd. (Zydus Cadila, Ahmedabad, India) was taken. Cost-effectiveness was calculated by dividing the cost of therapy by the change in DAS in a month. Adherence was assessed using the Morisky-Green-Levine Scale (MGLS) [11]. Patients were interviewed and asked to answer four questions listed in the questionnaire on their second visit. High adherence was denoted by a score of 0, medium adherence was denoted by a score of 1 or 2, and low adherence was denoted by a score of 3 or 4. The p value was taken to be 0.05. For the cost-effective analysis, the mean was applied to the DAS28 values and the cost of treatment. Statistical analysis was performed using the Statistical Package for the Social Sciences (SPSS) software (IBM SPSS Statistics, Armonk, NY, USA).

## Results

Table 1 represents the demographic profile of the patients. Out of 150 subjects, males and females were 21 (14%) and 129 (86%), respectively, with a median age of 48 years (range: 19-72 years). The median follow-up of the patients was in 9.4 weeks (range: 7-12 weeks). Of the patients, 34% had at least one associated comorbidity. Complementary alternative medicine (CAM) therapy was used by approximately 61% of the patients at least once. Monotherapy with one DMARD (methotrexate) was received by 110 (73.3%) participants, double DMARD therapy (methotrexate + hydroxychloroquine) was received by 35 (23.3%), triple DMARD therapy (methotrexate + hydroxychloroquine + leflunomide) was received by four (2.7%), and >3 DMARD therapy (methotrexate + hydroxychloroquine + leflunomide + biological DMARD adalimumab) was received by only one (0.7%). The mean DAS28 score decreased from 5.01 ± 1.19 to 4.42 ± 1.39 (p < 0.05) in the study cohort. Patients receiving either of four different combinations of DMARD therapy showed statistically significant improvement in terms of reduction in DAS28.

**Table 1:** Baseline demographic profile of the patients included in the study (N = 150) SD: standard deviation, DMARD: disease-modifying antirheumatic drug, CAM: complementary alternative medicine

Baseline demographics		
Gender distribution	Number (%)	
Males	21 (14)	
Females	129 (86)	
Age (in years)	Mean ± SD	Range
All patients	48.55 ± 11.89	19-72
Males	53.48 ± 9.21	33-70
Females	47.75 ± 12.11	19-72
Median age (in years)	48	
DMARD therapy received	Number (%)	
Monotherapy (methotrexate)	110 (73.3%)	
Double therapy (methotrexate + hydroxychloroquine)	35 (23.3%)	
Triple therapy (methotrexate + hydroxychloroquine + leflunomide)	4 (2.7%)	
Triple therapy with biological DMARD (methotrexate + hydroxychloroquine + leflunomide + adalimumab)	1 (0.7%)	
Associated comorbidities	51 (34%)	
CAM therapy	92 (61.4%)	
Median follow-up visit duration (in weeks)	9.4 (7-12)	

Cost of drug therapy

**Figure 1:**
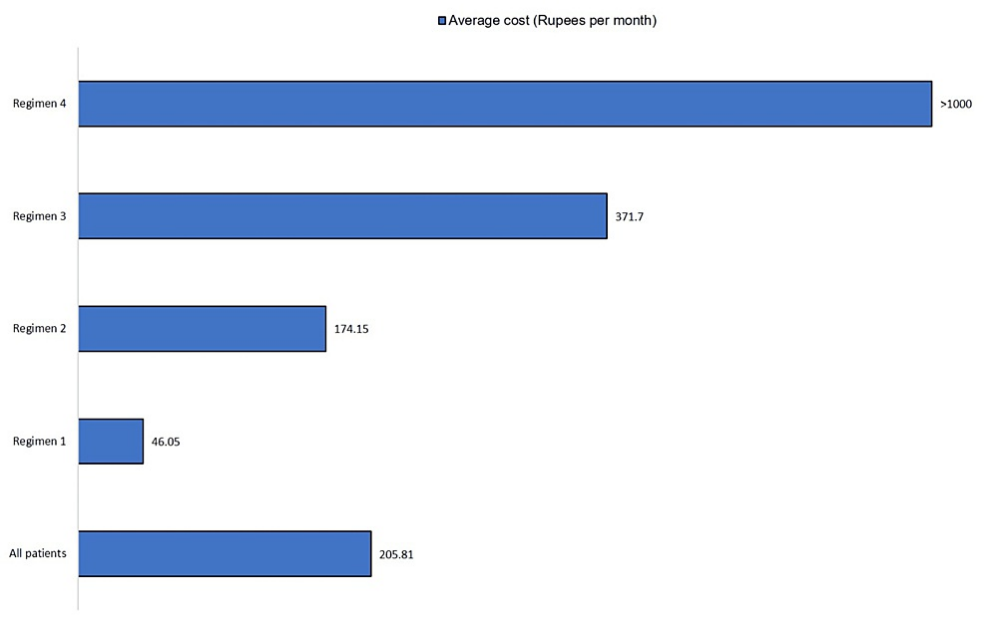
Direct medical cost of DMARDs in RA (N = 150) DMARDs: disease-modifying antirheumatic drugs, RA: rheumatoid arthritis

Figure 1 represents the direct cost of treatment in the management of rheumatoid arthritis in all patients and different regimens per month. Regimen 4 had the maximum cost of therapy (17,349.4 Rs).

Table 2 shows the cost-effectiveness calculation for the study participants. The mean cost per unit change in DAS28 was calculated.

**Table 2:** Cost-effective analysis for various DMARD therapy DMARD: disease-modifying antirheumatic drug, DAS: Disease Activity Score

	Mean DAS Baseline	Mean DAS at follow-up after one month	Change in DAS	Mean cost	Mean cost/change in DAS
Regimen 1	4.49	4.06	0.43	46.05	107.09
Regimen 2	6.25	5.27	0.98	174.15	177.70
Regimen 3	7.65	6.39	1.26	371.7	295
Regimen 4	8.04	7.38	0.66	17,349	26,287
Total	5.01	4.42	0.59	205.81	348.83

**Figure 2:**
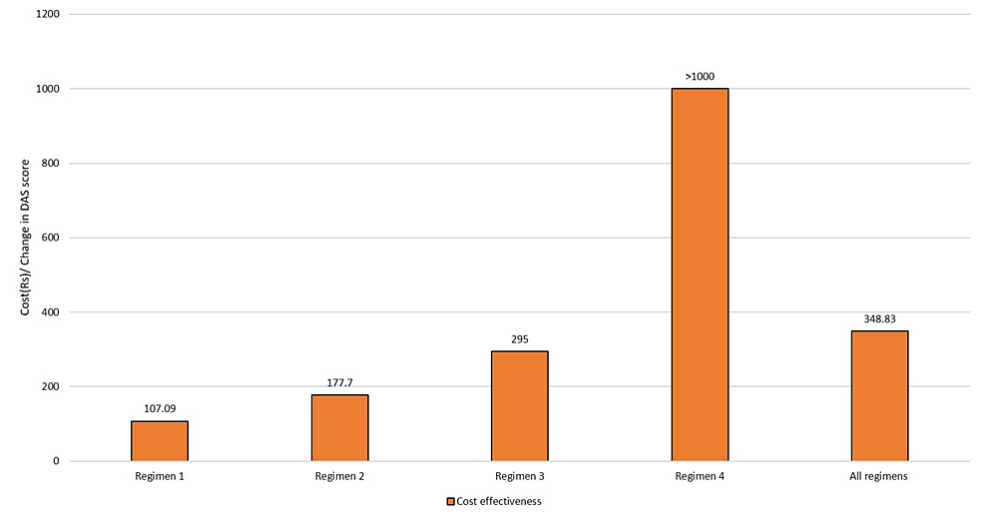
Comparative cost-effectiveness of the drug regimens DAS: Disease Activity Score

Figure 2 compares the cost-effectiveness of the regimens. The mean cost was the least with Regimen 1 with Rs 107.093 and the maximum with Regimen 4 with Rs 26,287.

Adherence

**Figure 3:**
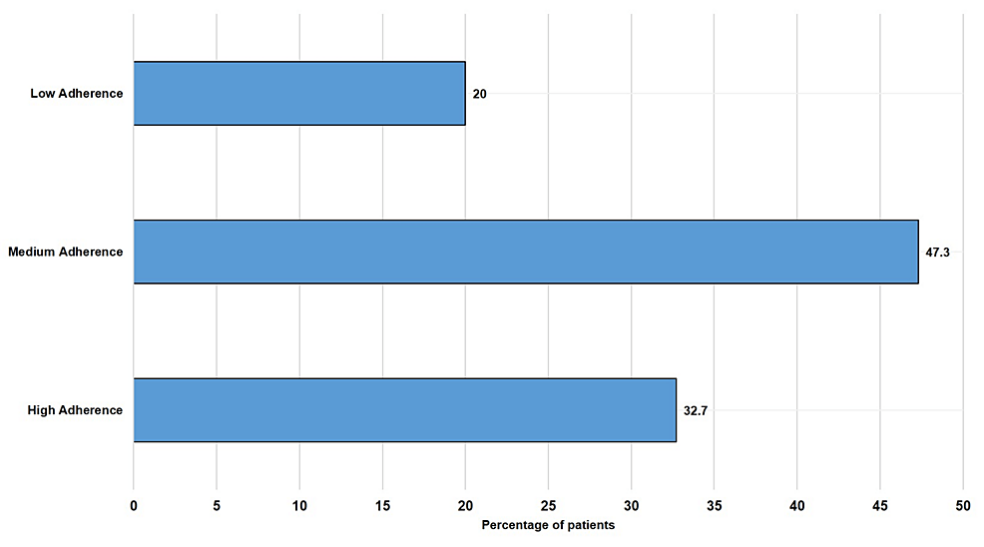
Level of adherence among the study population at follow-up (N = 150)

Figure 3 showcases adherence to therapy, which was calculated using the MGLS. At follow-up, the majority of the patients had medium adherence to drug therapy, followed by high adherence.

**Figure 4:**
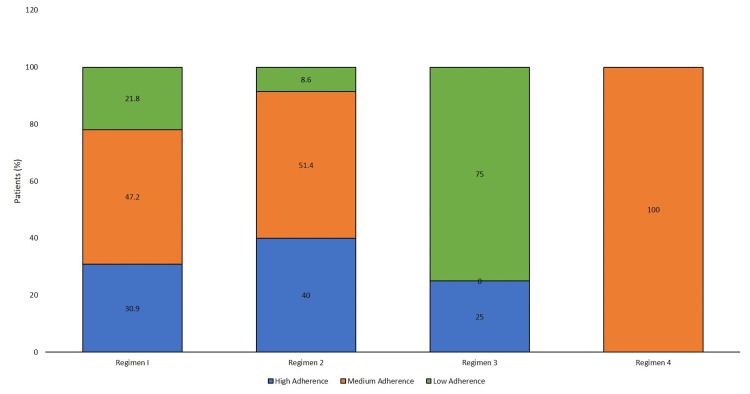
Adherence to DMARD therapy in patients receiving various prescription regimens at follow-up (N = 150) DMARD: disease-modifying antirheumatic drug

Figure 4 further elaborates on the percentage of patients showing the type of adherence within each drug regimen. The majority of patients receiving Regimen 1 (n = 110) and Regimen 2 (n = 35) revealed medium adherence (47.2% and 51.4%, respectively). However, within Regimen 3 (n = 4), low adherence was present in 75% of the patients. Only one patient receiving biological agents showed medium adherence.

On assessing the correlation of adherence to medication with DAS28 at baseline and follow-up visits, we obtained a negative correlation between DAS28 and adherence, where a higher DAS28 meant higher disease activity (r value: -0.1). However, the p values at both visits were not statically significant.

## Discussion

Cost-effectiveness

Our study makes an attempt to estimate the direct medical cost of DMARD therapy in a population of RA patients. In the present study, the overall mean cost of DMARD treatment was 205.81 Rs, with MTX (Regimen A) having the least cost of therapy with a mean of 46.05 Rs and MTX + HCQ + Lef + bDMARD adalimumab (Regimen D) having the maximum cost of therapy with a mean of 17,349.4 Rs.

Pharmacoeconomic studies on RA patients conducted in India are scanty. A study reported the mean direct cost of medical treatment per prescription of an RA patient to be 997.05 Rs monthly, of which the cost of medicine compromised 60.15%, laboratory and radiological investigations comprised 37.94%, and the consultation fee and ophthalmology costs comprised 1.91% [12].

Another such study concluded the mean direct cost of treatment of RA patients treated with synthetic DMARDs (sDMARDs) to be 623 ± 31 Rs monthly [13]. Yet another study calculated the mean direct cost of RA treatment with sDMARDs per month to be 696.57 ± 218.39 Rs ($15.92 ± $4.99) [11]. A different study carried out in a tertiary care center in Mumbai, India, estimated the mean total cost per prescription as 763.39 Rs [14].

In 2013, a certain study conducted in India estimated the direct cost of treatment with sDMARDs in RA as 1,929.99 Rs per month [15]. The cost of treatment reported in these studies is much higher as compared to our study. The possible reason for this could be that our study had only included the cost of DMARD agents. The cost calculation done in our study did not include concomitant medication, laboratory tests, radiological tests, and hospital consultation fees. Due to this reason, the results of the cost burden in the present study are far less when compared with other studies.

Few studies conducted outside India also assessed the comprehensive treatment cost of RA. A study done in Hong Kong on a biologic-naïve DMARD-treated Chinese population having RA calculated the yearly total costs for one patient to be US$9,286, out of which >60% was due to indirect costs such as work capacity/daily activity impairment [16]. Direct costs represented 11% of the total annual cost, comprising the patient’s “out-of-pocket” expenses and costs of inpatients, while a relatively small portion was attributed to the cost of medications [17].

Another study done in Japan [18] concluded that the average yearly cost of outpatient treatment of RA is US$2,705 for one patient. A different study done in Thailand [19] observed the mean direct and indirect costs for RA patients to be US$2,135 and US$547 per patient-year, respectively. The majority of pharmacoeconomic research studies are observed to have been carried out in western countries, most of them in the USA, the Netherlands, and Canada. According to a systematic review [20], the total mean costs for RA patients were estimated to be $18,725 yearly, which is almost twice that of the Hong Kong study [16]. In this review, the majority was made out of indirect costs contributing to 57% of the total costs.

Significant differences in the cost of DMARD therapy between studies were observed. Before the biologic therapy era, direct medical costs in RA were predominating due to hospitalization costs. Medication costs rose in proportion, making up the largest part of total therapy cost after the introduction of biologic therapy [21], due to the higher costs of biologic therapy [22]. Just as in our study, the maximum monthly average cost was contributed by one patient who was on Regimen 4, i.e., triple DMARD therapy with adalimumab. In the COBRA trial [23], treatment cost was assessed between RA patients on a combination of prednisolone, MTX, and sulfasalazine, and sulfasalazine alone. Here, the mean total costs per patient for approximately the first year were $5,519 for combined treatment and $6,511 for treatment with the stand-alone drug sulfasalazine.

Similar to our study, one study assessing cost economic analysis observed MTX monotherapy to be the least expensive ($12,842 for six months), while the combination of etanercept and MTX to be the most expensive ($19,083 for six months) [24]. When triple therapy was considered, the cost of the medicines comprised 33% of the direct costs, while medication costs increased substantially when considering etanercept monotherapy and etanercept + MTX, with 79 and 78% of the direct costs, respectively [17]. A study done in Japan concluded the mean total actual healthcare monthly cost for patients on biologics to be 26,636 Rs (US$409.7) per month per patient, which correlated with the treatment cost seen in Regimen 4 in our study. The results of the methotrexate group were 1,936 Rs (US$29.79) monthly in the Japanese study, while they were comparatively lower in Regimen 1 in our study [25].

Variability in the cost of treatment may be a result of multiple factors such as the severity of the disease, a variable number of patients, usage of different combinations of DMARDs having varying costs, different methods of cost computation, and the differences in healthcare approach in different parts of the world. One more important reason for low-cost therapy from other Indian studies can also be attributed to the use of generic medicines in our hospital, which significantly reduces the cost of therapy.

Cost-effective analysis with the outcome as the DAS revealed that the overall cost-effectiveness with DMARD therapy was 878.14 Rs per unit change of DAS28. Regimen 1 was the most cost-effective treatment with the least cost of 290.9 Rs for a unit change of DAS28, and the least cost-effective was Regimen 4 with 65,661.8 Rs for a unit change of DAS28. Although the absolute values of treatment cost were different from other studies, the results are in concurrence with the review of the cost-effectiveness of RA treatments and the clinical recommendations of the European League Against Rheumatism (EULAR). These findings endorse the view that traditional DMARDs are cost-effective and useful in controlling disease activity at the time of disease onset [26]. The majority of the studies assessing cost-effectiveness reported outcome results as an incremental cost-effectiveness ratio (ICER) and quality-adjusted life year (QALY) gained by patients. The follow-up duration in these studies ranged between 24 and 240 weeks. This was in contrast to our study where the outcome was reported in terms of change in DAS as the follow-up of the patients was done at one month.

Adherence scale

For any pharmacological intervention to be a success, proper adherence to therapy is of much importance [27]. A study assessing medication adherence to DMARDs reported adherence ranging between 30% and 107% [28]. In the present study, the MGLS was used, in which the majority of patients showed medium adherence. This pattern was common in patients receiving monotherapy and dual-drug therapy. In triple therapy, low adherence was the predominant pattern. According to questions asked in the MGLS, many people responded to having been careless with taking medication; some stopped taking when they felt better, thus interfering with the proper completion of therapy; and some admitted to having become irregular users if they felt no improvement in pain, stiffness, or restriction of movement. All these factors contributed to the widely different patterns in adherence therapy. Our results were in concurrence with a 2018 study done in Austria, which stated a similar reason “largely due to forgetting to intake medication” for lower medication adherence [29].

A study done in Texas, USA, in 2013 stated that patients with RA who had lower disease activity were shown to have better adherence than patients with opposite characteristics [30]. In our study, also, adherence to medication with DAS28 revealed a negative correlation, although the p values were not statically significant. In a 2022 study done in Japan for patients with RA on MTX, based on the Eight-Item Morisky Medication Adherence (MMAS-8) scale, medium adherence was seen in most of the patients (60%), followed by high adherence (27.9%), and then low adherence (12.1%), which was in concurrence with our results [31].

The limitations of our study were that RA being a chronic disease that often spans decades, the follow-up duration of 12 weeks might have limited some findings. Moreover, since this was an observational study, the number of patients in each of the four drug regimen groups was not equal, which may have led to some confounding bias in the results. The results of this study can further be validated with some randomized double-blind studies having a longer follow-up period.

## Conclusions

Our study concludes that the cost burden varied according to the number of DMARDs being given to the patient, so the more severe the disease, the more drugs were given and the more was the direct cost burden.

On a whole, the majority of patients had “medium adherence” to therapy, with those on the single drug MTX having the maximum high adherence.
